# Time Series Analysis of Climate and Air Pollution Factors Associated with Atmospheric Nitrogen Dioxide Concentration in Japan

**DOI:** 10.3390/ijerph17249507

**Published:** 2020-12-18

**Authors:** Takeshi Miyama, Hiroshi Matsui, Kenichi Azuma, Chika Minejima, Yasuyuki Itano, Norimichi Takenaka, Masayuki Ohyama

**Affiliations:** 1 Division of Public Health, Osaka Institute of Public Health, Osaka 537-0025, Japan; 2 Division of Hygienic Chemistry, Osaka Institute of Public Health, Osaka 537-0025, Japan; matsuih@iph.osaka.jp (H.M.); ohyama@iph.osaka.jp (M.O.); 3 Department of Environmental Medicine and Behavioural Science, Faculty of Medicine Kindai University, Osakasayama 589-8511, Japan; kenazuma@med.kindai.ac.jp; 4 Department of Natural Sciences, College of Liberal Arts, International Christian University, Mitaka 181-8585, Japan; minejimachika@gmail.com; 5 Osaka City Research Center of Environmental Science, Osaka 543-0026, Japan; y-itano@city.osaka.lg.jp; 6 Department of Applied Chemistry, Graduate School of Engineering, Osaka Prefecture University, Sakai 599-8531, Japan; takenaka@chem.osakafu-u.ac.jp

**Keywords:** air pollution, climate, HONO, Japan, NO_2_, time series analysis

## Abstract

Nitrogen dioxide (NO_2_) is an air pollutant discharged from combustion of human activities. Nitrous acid (HONO), measured as NO_2_, is thought to impact respiratory function more than NO_2_. HONO and NO_2_ have an equilibrium relationship, and their reaction is affected by climate conditions. This study was conducted to discuss the extent of HONO contained in NO_2_, depending on the level of urbanization. Whether climate conditions that promote HONO production enhanced the level of NO_2_ measured was investigated using time series analysis. Climate and outdoor air pollution data measured in April 2009–March 2017 in urban (Tokyo, Osaka, and Aichi) and rural (Yamanashi) areas in Japan were used for the analysis. Air temperature had a trend of negative associations with NO_2_, which might indicate the decomposition of HONO in the equilibrium between HONO and NO_2_. The associations of relative humidity with NO_2_ did not have consistent trends by prefecture: humidity only in Yamanashi was positively associated with NO_2_. In high relative humidity conditions, the equilibrium goes towards HONO production, which was observed in Yamanashi, suggesting the proportion of HONO in NO_2_ might be low/high in urban/rural areas.

## 1. Introduction

Nitrogen dioxide (NO_2_) is an air pollutant discharged from combustion of human activities such as driving cars, and numerous epidemiological studies have suggested a relationship between NO_2_ and impaired respiratory function or asthma symptoms [1,2,3,4].

NO_2_ is monitored at air pollution monitoring stations (APMSs) in Japan [5], and previous studies have implied that climate factors affect NO_2_ levels [1,6,7]. Since climate and air pollutant time series data demonstrate seasonality and autocorrelation effects, those effects should be adjusted to find associations among the factors. A large number of studies investigated the effect of NO_2_ on human health status such as morbidity and mortality of diseases using time series analysis (e.g., [8,9,10]). In those studies, climate and other pollutant factors were dealt as confounders/covariates of NO_2_ to control (i.e., an outcome, exposure, and confounders/covariates were the number of cases, NO_2_, and climate/other pollutant factors, respectively), indicating they are associated with NO_2_ concentration. However, studies that directly estimate the relationships between climate/co-pollutant factors and NO_2_ using time series analysis are limited.

In the environment, nitrous acid (HONO) is in equilibrium with NO_2_, nitric oxide (NO) and H_2_O (Equation (1)). It is reported that this is the main chemical reaction for HONO production [11,12]:

(1) $$2\mathrm{HONO}⇄\mathrm{NO}+{\mathrm{NO}}_{2}+H_{2}O$$

The direction of this reaction depends on climate conditions: high air temperature promotes NO_2_ production since the forward reaction is endothermic, and high humidity promotes HONO production since an abundance of H_2_O promotes the reverse reaction [7,13], in which the effects on HONO and NO_2_ production are also reversed. The other characteristics differ between HONO and NO_2_ with climate conditions are water solubility and decomposition by sunlight. HONO is much more water-soluble [12] and is more easily decomposed by sunlight [7] than NO_2_. Moreover, it has been found that conventional assays of NO_2_ such as the Saltzmann reagent method and NOx analyzer measure HONO as NO_2_ [14]. A few studies have suggested that HONO impacts respiratory function more than either NO_2_ or sulfur dioxide alone [15], and that the main sources of indoor HONO are indoor combustion and outdoor NO_2_ [6]. The World Health Organization [16] reported that it is unclear to what extent the health effects observed in epidemiological studies are attributable to NO_2_ itself, or rather to primary and secondary combustion-related products (i.e., organic carbon and HONO) with which it is typically correlated. However, it is argued that the available scientific literature has not accumulated sufficient evidence to justify revising the existing WHO air quality guidelines for annual NO_2_ concentrations. To clarify the effects of NO_2_ and HONO, monitoring the concentrations of environment HONO at APMSs is desired; however, these concentrations are not currently measured.

In the equilibrium reaction (Equation (1)), HONO production is promoted by a temperature decline and humidity rise, and vice versa for NO_2_. Thus, the associations of climate factors with NO_2_ might indicate the effects of these factors on either NO_2_ or HONO. This study was conducted to investigate the relationships between climate factors and NO_2_ concentration measured at APMSs using time series analyses. Then, from the main results, the level of HONO (measured as a part of NO_2_) in NO_2_ concentration was discussed.

## 2. Materials and Methods

### 2.1. Study Area

Tokyo, Osaka, Aichi, and Yamanashi Prefectures were chosen as study areas. Tokyo, Osaka and Aichi were chosen as urban study sites. These prefectures include the three largest cities in Japan, the special wards of Tokyo, Osaka City, and Nagoya City, and are highly populated (first, third, fourth highest populations out of 47 prefectures in Japan in 2015, respectively) [17]. Yamanashi was selected as a rural study area and has a lower population (the seventh lowest out of 47 prefectures) and is located around the mid-point between Tokyo and Aichi.

### 2.2. Data Collection

Time series data of outdoor air pollution and climate factors between April 2009 and March 2017 were used in this study. From each prefecture studied, the roadside APMS (RAPMS) that had the highest monthly NO_2_ emission in September 2009 and the closest ambient air pollution monitoring station (AAPMS) from each RAPMS was selected, and hourly outdoor air pollutant concentration data were collected from the monitoring stations [5]. The air pollutants consisted of NO_2_ (ppb), nitric oxide (NO, ppb), and ozone (O_3_, ppb). If data were missing for three consecutive hours, the missing data were kept as missing, otherwise they were imputed by linear interpolation. Each daily datum was calculated as the average of the hourly data of that day unless they contained missing data. Then, all daily missing data were imputed further by linear interpolation. The numbers of missing hourly and daily data by the compounds and APMSs are shown in Table S1. The concentration of active O_3_ (AO) was defined as the difference in concentrations between potential ozone (PO) and NO_2_ at the RAPMS. PO was calculated as the sum of NO_2_ and O_3_ at the AAPMSs. AO was used as the alternative of O_3_ at the RAPMS for the analysis below because the concentration of O_3_ is negligibly low at roadside due to high consumption by reactions with NO and others. Daily climate data were also collected from the meteorological station [18] in each prefecture that was the closest to the air pollution monitoring stations. The climate data included the mean air temperature (°C), relative humidity (%), mean wind speed (m/s), total duration of sunshine (hour), and mean global solar radiation (MJ/m^2^). The average monthly concentrations of outdoor pollutants and the average or total monthly values of climate factors were calculated from the data collected. All the data used were publicly available, and no ethical approval was required.

### 2.3. Statistical Analysis

#### 2.3.1. Descriptive Analysis

Monthly NO_2_ concentration dynamics by monitoring stations and regions were plotted to assess NO_2_ levels for the type of monitoring stations and for the regions.

#### 2.3.2. Time Series Prediction Model for NO_2_ (SARIMAX Model)

The time series data were divided into training (April 2009–March 2016) and testing (April 2016–March 2017) data to allow for cross-validation. With the training data, time series models for monthly NO_2_ concentrations were built using seasonal autoregressive integrated moving average (SARIMA) and SARIMA with exogeneous variable (SARIMAX) models [19]. These models were built to assess the relationships between NO_2_ and the climate factors/outdoor air pollutants after removing the effects of autocorrelation and seasonality which are commonly contained in time series data. The logarithm of monthly average NO_2_ concentration was used as a response variable. One of the climate factors/air pollutants was added as an exogeneous variable for each model. In total, 64 models were built for the four regions, two monitoring stations, and eight exogeneous variables (including non-exogeneous (SARIMA) models). A SARIMA model is an extension from an ARIMA model to which the seasonal terms are added. An ARIMA model consists of non-seasonal autoregressive and moving average terms and were transformed to stationary from non-stationary series by differencing. The relationships between NO_2_ and exogeneous variables can be evaluated by adjusting the coefficient of the exogeneous variables given the autocorrelation terms are included in the models. For the non-seasonal terms, the number of previous time series used for autoregression and moving averaging, and the number of differencing to reach stationary series were described as p, d, and q, while those for seasonal terms were P, D, and Q, respectively.

Time series analysis was conducted using the Box-Jenkins approach [20]. The Box-Jenkins approach contains model stabilization, identification, model validation, forecasting and cross-validation. For model stabilization and identification, the Hyndman-Khandakar algorithm [21] for automatic ARIMA modelling was used, which is available in the “forecast” package in R as the “auto.arima” function, to select the best model based on the Akaike Information Criterion (AIC, the lowest AIC models were selected as the best models). The Ljung-Box tests were then used to test the randomness of the residuals for the model validation. If the model failed to pass the Ljung-Box test, differenced data were used to select the best fit model based on AIC [19] using the “auto.arima” function. Finally, using the testing data, a forecasting for the preceding 12 months was conducted. The model fitting and forecasting were evaluated using the root mean squared errors (RMSEs). All the analyses were performed using Statistical Software R version 4.0.0 [22].

## 3. Results

### 3.1. Descriptive Analysis (NO_2_ Dynamics in April 2009–March 2017)

**Figure 1:**
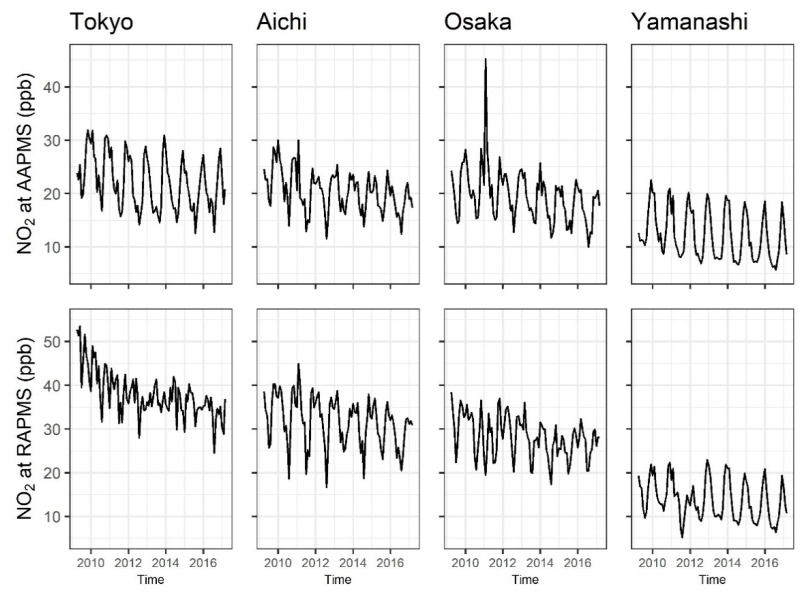
Monthly average nitrogen dioxide (NO_2_) concentration in April 2009–March 2017 in the study areas, Tokyo, Aichi, Osaka, and Yamanashi, Japan. Upper figures are NO_2_ concentrations at ambient air pollution monitoring stations (AAPMSs) while lowers are at roadside air pollution monitoring stations (RAPMSs).

Among the four study areas, Tokyo had the highest NO_2_ concentration both at AAPMS and RAPMS, and Yamanashi has the lowest (Table 1 and Figure 1). The dynamics of NO_2_ had annual seasonality showing the highest/lowest concentrations in summer (August)/winter (basically November–February), respectively; however, the seasonality at RAPMS was less clear than that at AAPMS (Table 1).

### 3.2. Climate and Air Pollution Factors Associated with Nitrogen Dioxide (NO_2_) Concentration at Ambient Air Pollution Monitoring Station (AAPMS)

Overall, air temperature, wind speed, solar radiation, and O_3_ at AAPMS had a trend of negative associations with NO_2_, while NO had positive associations (Table 2, the result of model validations is available in Table S2).

**Figure 2:**
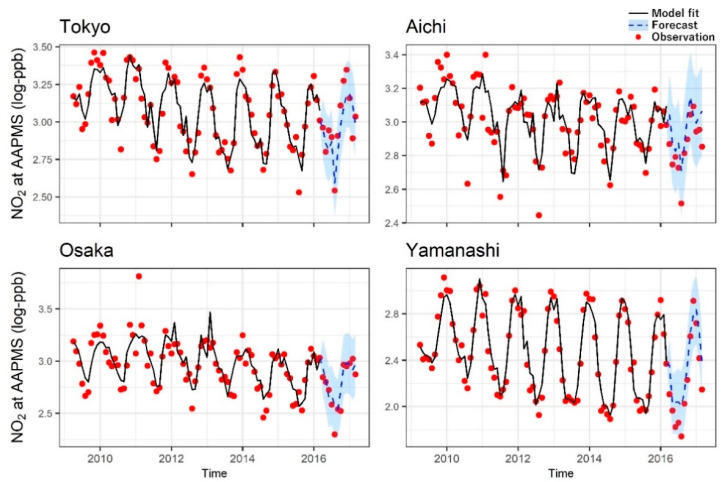
Fitting and forecasting for nitrogen dioxide (NO_2_) concentration at ambient air pollution monitoring stations (AAPMSs) using seasonal autoregressive integrated moving average (SARIMA) models. Log scale NO_2_ concentrations were used for the analyses. Red dots are observations. Black lines show model fits for NO_2_ using data in April 2009–March 2016 (training data). Blue dotted lines (estimates) and shadows (95% confidence intervals) show the forecasting for NO_2_ using data in April 2016–March 2017 (testing data).

Specifically, air temperature had significant negative associations with NO_2_ in all the prefectures except Yamanashi. Wind speed had negative associations with NO_2_ in all the prefectures. Solar radiation had negative associations with NO_2_ in all the prefectures except Osaka. O_3_ was negatively and significantly associated with NO_2_ only in Yamanashi. NO had positive associations with NO_2_ in all the prefectures. The associations of relative humidity and sunshine duration with NO_2_ did not have consistent trends by prefecture (Table 2). Humidity in Osaka was negatively associated with NO_2_ while that in Yamanashi was positively associated. Sunshine duration had a trend of negative association with NO_2_ except in Osaka; Yamanashi had a significant negative association, while Osaka had a positive association. RMSEs for forecasting were close to those for model fitting (Table S2), and most of the testing data were within the 95% confidence interval (CI) of the forecasting (see Figure 2 as examples).

### 3.3. Climate and Air Pollution Factors Associated with Nitrogen Dioxide (NO_2_) Concentration at Roadside Air Pollution Monitoring Station (RAPMS)

Air temperature, wind speed, NO, and AO (the alternative of O_3_) at RAPMS had similar trends as those at AAPMS, and the first two were negatively associated with NO_2_, while NO was positively associated (Table 2, the result of model validations is available in Table S3). Specifically, air temperature had significant negative associations with NO_2_ in all the prefectures except Tokyo. Wind speed had negative associations with NO_2_ in all the prefectures. NO had positive associations with NO_2_ in all the prefectures. AO had significant negative associations with NO_2_ in Osaka and Yamanashi. The associations of relative humidity with NO_2_ did not have a consistent trend by prefecture (Table 2) as observed at AAPMS. The trend of relative humidity at RAPMS was the same as that at AAPMS: in Osaka it was negatively associated with NO_2_ while in Yamanashi it was positively associated. Sunshine duration had a trend of positive association with NO_2_ except in Osaka; significant associations were observed in Tokyo and Osaka at RAPMS. No significant relationship between solar radiation and NO_2_ was observed at RAPMS.

**Figure 3:**
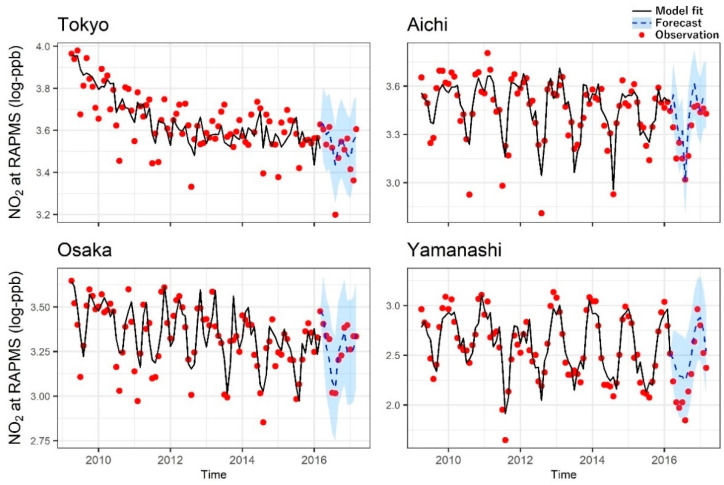
Fitting and forecasting for nitrogen dioxide (NO_2_) concentration at roadside air pollution monitoring stations (RAPMSs) using seasonal autoregressive integrated moving average (SARIMA) models. Log scale NO_2_ concentrations were used for the analyses. Red dots are observations. Black lines show model fits for NO_2_ using data in April 2009–March 2016 (training data). Blue dotted lines (estimates) and shadows (95% confidence intervals) show the forecasting for NO_2_ using data in April 2016–March 2017 (testing data).

RMSEs for forecasting were close to those for model fitting (Table S3), and most of the testing data were within the 95% CI of the forecasting (see Figure 3 as examples, showing SARIMA model fittings and forecastings).

## 4. Discussion

NO_2_ air pollutant concentration shows seasonality, that is, low in summer and high in winter. The seasonality of NO_2_ at RAPMS was not clear compared to that at AAPMS, especially in urban areas (Tokyo, Aichi, and Osaka, i.e., NO_2_ levels at AAPMSs had regular annual patterns as shown in Figure 1 while those at RAPMSs in urban areas had irregular shape). Although the reasons for this are not clear, it might be affected by the emission of air pollutants from road traffic. According to the Ministry of Land, Infrastructure, Transport and Tourism, Japan [23], the average traffic volume of measuring points within 2 square kilometres of the centre of the RAPMSs in Tokyo, Aichi, and Osaka were 53,892, 51,894, and 43,004 cars per day in 2015, respectively, and the volumes were higher than in Yamanashi at 13,910 cars per day. NO_2_ was high in the urban areas and low in the rural area. The traffic volume could be one of the factors that affects NO_2_ concentrations in the atmosphere.

Air temperature, wind speed, and solar radiation at AAPMSs had a trend of negative association with NO_2_, while NO had a positive association (Table 2). The effect of solar radiation and wind speed on HONO and NO_2_ are consistent. HONO is decomposed by solar light [7], and the effect of decomposition by sunlight for NO_2_ is much weaker [24]. Wind decreases the concentrations of NO_2_ and HONO in the atmosphere [1,7]. When considering the relationships between climate factors and NO_2_ concentration in the atmosphere, the equilibrium relationship (Equation (1)) can also be considered. Given a high air temperature, the reaction proceeds toward the right side (i.e., producing NO_2_), while it goes to the left side (producing HONO) given high humidity [7]. Although the proportion of HONO out of NO_2_ is a few percent, the change in HONO concentration must be larger than NO_2_ from the equilibrium Equation (1) (i.e., one molecule of NO_2_ produces two HONO molecules). The negative relationship between air temperature and the NO_2_ concentration measured at AAPMS from this study suggests that the identified associations showed the same trends for HONO with climate factors, and that a certain proportion of HONO was in measured NO_2_.

The associations of relative humidity and sunshine duration with NO_2_ did not have consistent trends by prefecture. In the case of relative humidity, the relationship observed in Yamanashi seemed to be an effect of humidity on HONO in terms of the equilibrium relationship between HONO and NO_2_ (Equation (1)), while that in Osaka was an effect on NO_2_. Urban areas are more polluted by NO/NO_2_ compared to rural areas due to higher amounts of combustion, and the level of HONO does not reach an equilibrium concentration. Because of this, it is suggested that the proportion of HONO in measured NO_2_ was low/high in urban/rural areas, respectively, and the change in HONO concentration could be more sensitive in rural areas. This idea is consistent with a report saying the HONO/NO_x_ ratio at roadsides was low [25], while that indoors, which may correlate with the ambient HONO/NO_x_ concentrations, was high [6]. As described in (Equation (1)), HONO is produced from NO and NO_2_ discharged from, for example, cars. This reaction takes time to reach the equilibrium status [26], and it indicates that rural areas are polluted by HONO not locally but diffusely.

The trends of association of climate factors/air pollutants with NO_2_ at RAPMS were almost consistent with those at AAPMS although a significant relationship between solar radiation and NO_2_ was not observed at RAPMS. The lower effects of solar radiation on NO_2_ measured at RAPMSs suggested a higher proportion of NO and NO_2_ concentration due to their higher emission, thus, a lower proportion of HONO could be considered at RAPMS than at AAPMS (i.e., the level of HONO had not reached the equilibrium concentration at RAPMSs) because the effect of decomposition by sunlight for NO_2_ is much weaker than that for HONO [24]

Correlations between NO_2_ and O_3_ previously reported vary depending on the temporal averaging periods (i.e., from within-hourly to annual or longer-term correlations) and also depending on the studies [1]. One study using monthly averaging period, which is the same averaging period as the current study, showed similar results to this study (i.e., negative correlation between NO_2_ and O_3_). O_3_ is consumed by reactions with NO producing NO_2_ ($\mathrm{NO}+O_{3}\to {\mathrm{NO}}_{2}+O_{2}\;$), and its concentration can be negligibly low due to high consumption where NO concentration is high. The negative relationship between O_3_ and NO_2_ from this study can be explained by this reaction and the extent of O_3_ concentrations, which was relatively low. The relationships between NO_2_ and O_3_ concentrations may have seasonal patterns, although the evidence is limited [1]; however, the present study did not deal with the effect modification of seasons, and further studies are required to assess the interaction effect between O_3_ and seasons on NO_2_ concentrations.

SARIMAX models can estimate coefficient of exogeneous variables by adjusting autocorrelation and seasonality effects. However, in this study only one exogeneous variable was used for each SARIMA model for the estimation. It should be noted that other confounders were not adjusted because underlying causal relationships between factors were not well established, and data used were limited to climate factors and air pollutants.

## 5. Conclusions

The relationships between climate factors and NO_2_ concentrations measured in the atmosphere, the differences between the types of monitoring stations, and differences between urban and rural areas were assessed using SARIMA and SARIMAX models in this study. Overall, wind speed was negatively and NO was positively associated with NO_2_ concentration. Air temperature had a negative association with NO_2_, which might indicate the decomposition of HONO (that was in measured NO_2_) in the equilibrium (Equation (1)). Humidity in Osaka was negatively associated with NO_2_ (the reaction of NO_2_) while that in Yamanashi was positively associated (the reaction of HONO), indicating the proportion of HONO in NO_2_ might be low/high in urban/rural areas, respectively. The associations between solar radiation and NO_2_ concentrations were only observed at the AAPMSs where the proportion of HONO in NO_2_ measured might have been higher. The findings from this study may suggest that the equilibrium reaction of HONO had a large impact against the ambient NO_2_ concentrations. To test the hypotheses that arose from this study (i.e., the level of HONO in NO_2_ in different settings), monitoring of HONO concentration and further analyses of the relationship between HONO and climate/pollutant factors are required.

## Figures and Tables

**Table 1:** Mean (standard deviation) NO_2_ concentration (ppb) by month between April 2009 and March 2017 at AAPMSs and RAPMSs in Tokyo, Aichi, Osaka, and Yamanashi Prefectures, Japan.

	AAPMS				RAPMS			
Month	Tokyo	Aichi	Osaka	Yamanashi	Tokyo	Aichi	Osaka	Yamanashi
January	26.5 (2.0)	22.2 (3.5)	22.9 (2.9)	17.8 (1.6)	34.5 (3.1)	34.3 (2.2)	28.0 (3.5)	18.5 (3.0)
February	25.2 (4.1)	23.0 (3.6)	24.6 (8.6)	16.3 (3.0)	37.9 (6.2)	36.7 (4.2)	28.6 (4.7)	17.8 (3.2)
March	23.1 (2.4)	21.9 (2.9)	22.5 (3.0)	12.3 (2.7)	38.8 (3.7)	36.2 (3.7)	30.9 (3.5)	14.4 (2.5)
April	21.1 (2.7)	21.0 (2.3)	20.8 (2.7)	10.6 (1.6)	41.5 (5.8)	33.6 (3.0)	32.1 (3.5)	13.3 (3.0)
May	18.8 (2.2)	18.4 (2.3)	18.7 (1.7)	8.7 (1.6)	39.4 (5.4)	29.5 (3.5)	29.5 (3.7)	11.6 (3.3)
June	19.6 (3.6)	19.0 (2.5)	17.4 (2.9)	8.7 (2.2)	42.4 (5.0)	29.0 (2.8)	28.1 (2.9)	11.2 (3.1)
July	18.2 (1.1)	16.7 (2.0)	16.1 (2.1)	8.1 (1.5)	37.2 (3.4)	24.7 (2.3)	21.7 (1.8)	9.5 (2.0)
August	15.2 (2.3)	14.4 (2.0)	13.8 (2.2)	7.7 (1.4)	32.7 (6.3)	21.8 (3.7)	21.1 (2.6)	8.4 (2.0)
September	18.2 (3.6)	18.0 (2.8)	14.3 (1.6)	8.8 (1.7)	37.6 (6.5)	28.3 (4.0)	26.6 (2.9)	9.9 (1.7)
October	22.5 (4.9)	22.2 (3.5)	17.6 (3.8)	12.1 (2.4)	38.6 (4.8)	34.8 (4.3)	30.5 (4.2)	12.7 (2.1)
November	27.8 (3.0)	24.4 (2.1)	23.3 (3.3)	16.9 (2.5)	38.2 (5.0)	37.2 (2.8)	32.5 (3.4)	17.7 (2.7)
December	28.9 (1.8)	23.1 (2.2)	22.8 (2.5)	19.5 (1.9)	36.8 (2.7)	34.2 (1.7)	29.0 (2.9)	20.1 (3.0)

NO_2_, nitrogen dioxide; AAPMSs, ambient air pollution monitoring stations; RAPMSs, roadside air pollution monitoring stations.

**Table 2:** Climate and air pollution factors associated with NO_2_ concentration in the atmosphere from SARIMAX models at AAPMSs/RAPMSs in Tokyo, Aichi, Osaka, and Yamanashi Prefectures, Japan.

Model ^1^ (SARIMA/ SARIMAX)	AAPMS	RAPMS
	Coefficient ^2^ (95%CI)	
Tokyo		
SARIMA	NA	NA
Temperature	−0.022 (−0.029, −0.015)	−0.001 (−0.005, 0.003)
Humidity	0.004 (−0.001, 0.008)	0.002 (−0.001, 0.004)
Wind speed	−0.181 (−0.242, −0.120)	−0.297 (−0.344, −0.251)
Solar radiation	−0.021 (−0.033, −0.009)	−0.002 (−0.01, 0.005)
Sunshine duration	−0.018 (−0.037, 0.001)	−0.021 (−0.039, −0.003)
NO	0.024 (0.016, 0.031)	0.006 (0.005, 0.008)
O_3_/AO ^3^	−0.003 (−0.009, 0.002)	−0.003 (−0.012, 0.001)
Aichi		
SARIMA	NA	NA
Temperature	−0.015 (−0.020, −0.009)	−0.016 (−0.022, −0.009)
Humidity	−0.003 (−0.010, 0.004)	−0.005 (−0.010, 0.000)
Wind speed	−0.270 (−0.343, −0.196)	−0.169 (−0.238, −0.100)
Solar radiation	−0.019 (−0.031, −0.007)	−0.002 (−0.014, 0.011)
Sunshine duration	−0.013 (−0.034, 0.008)	−0.002 (−0.020, 0.016)
NO	0.020 (0.013, 0.028)	0.009 (0.007, 0.012)
O_3_/AO ^3^	−0.001 (−0.008, 0.005)	0.001 (−0.005, 0.007)
Osaka		
SARIMA	NA	NA
Temperature	−0.022 (−0.026, −0.018)	−0.011 (−0.013, −0.008)
Humidity	−0.010 (−0.019, −0.001)	−0.006 (−0.012, −0.001)
Wind speed	−0.306 (−0.413, −0.199)	−0.257 (−0.317, −0.198)
Solar radiation	0.002 (−0.025, 0.029)	0.01 (−0.006, 0.026)
Sunshine duration	0.061 (0.031, 0.091)	0.025 (0.003, 0.047)
NO	0.033 (0.027, 0.039)	0.015 (0.012, 0.018)
O_3_/AO ^3^	−0.002 (−0.009, 0.005)	−0.006 (−0.011, −0.001)
Yamanashi		
SARIMA	NA	NA
Temperature	−0.012 (−0.034, 0.010)	−0.031 (−0.038, −0.024)
Humidity	0.010 (0.006, 0.013)	0.005 (0.001, 0.010)
Wind speed	−0.198 (−0.251, −0.144)	−0.120 (−0.208, −0.032)
Solar radiation	−0.016 (−0.029, −0.004)	−0.005 (−0.022, 0.012)
Sunshine duration	−0.025 (−0.043, −0.006)	0.012 (−0.008, 0.032)
NO	0.034 (0.021, 0.047)	0.038 (0.025, 0.050)
O_3_/AO ^3^	−0.007 (−0.012, −0.002)	−0.009 (−0.015, −0.005)

*Notes*: The first 7 years [April 2009–March 2016, 84-point (12 months times 7 years) time series data] were used as training data and the last 1 year [April 2016–March 2017, 12-point (12 months times 1 year) time series data] were as training data for each model. AAPMS, ambient air pollution monitoring stations; RAPMS, roadside air pollution monitoring stations; SARIMA, seasonal autoregressive integrated moving average; SARIMAX, seasonal autoregressive integrated moving average with exogeneous variables; CI, confidence interval; NO_2_, nitrogen dioxide; NO, nitric oxide; O_3_, ozone. ^1^ This column shows the types of models. The exogeneous variables are shown for SARIMAX models. An exogeneous variable was included in each SARIMAX model. ^2^ Coefficients in log scale for exogeneous variable. ^3^ O_3_/AO were used for the analyses at AAPMSs/RAPMSs, respectively.

## Supplementary Materials

The following is available online at https://www.mdpi.com/1660-4601/17/24/9507/s1, Table S1: The numbers of missingness of hourly and daily data by the compounds and air pollution monitoring stations; Table S2: Climate and air pollution factors associated with NO_2_ concentration in the atmosphere from SARIMAX models at AAPMSs in Tokyo, Aichi, Osaka, and Yamanashi Prefectures, Japan; Table S3: Climate and air pollution factors associated with NO_2_ concentration in the atmosphere from SARIMAX models at RAPMS in Tokyo, Aichi, Osaka, and Yamanashi Prefectures, Japan.
