# Supplementary material for: Time Series Analysis of Climate and Air Pollution Factors Associated with Atmospheric Nitrogen Dioxide Concentration in Japan
Source: Int J Environ Res Public Health. 2020 Dec 18;17(24):9507. doi: 10.3390/ijerph17249507 (PMC7767269; doi:10.3390/ijerph17249507)
Supplement: Supplementary file 1 [file ijerph-17-09507-s001.pdf]

Supplemental material for: Time Series Analysis of Climate and Air Pollution Factors Associated with Atmospheric Nitrogen Dioxide Concentration in Japan

Takeshi Miyama, Hiroshi Matsui, Kenichi Azuma, Chika Minejima, Yasuyuki Itano, Norimichi Takenaka and Masayuki Ohyama

Table S1. The numbers of missingness of hourly and daily data (out of 70,152 and 2,923, respectively) by the compounds and air pollution monitoring stations.

	NO	NO_2	O_3
**Hourly data**			
AAPMS			
Tokyo	2561	2561	2612
Aichi	1423	1423	1058
Osaka	1901	1901	1328
Yamanashi	1121	1121	2430
RAPMS			
Tokyo	1392	1392	Not Applicable
Aichi	1234	1234	Not Applicable
Osaka	1061	1061	Not Applicable
Yamanashi	3303	3303	Not Applicable
**Daily data**			
AAPMS			
Tokyo	94	94	102
Aichi	51	51	48
Osaka	83	83	58
Yamanashi	54	54	76
RAPMS			
Tokyo	34	34	Not Applicable
Aichi	38	38	Not Applicable
Osaka	51	51	Not Applicable
Yamanashi	155	155	Not Applicable

Notes: AAPMS, ambient air pollution monitoring station; RAPMS, roadside air pollution monitoring station; NO_2, nitrogen dioxide; NO, nitric oxide; O_3, ozone

Table S2. Climate and air pollution factors associated with NO_2 concentration in the atmosphere from SARIMAX models at AAPMSs in Tokyo, Aichi, Osaka, and Yamanashi Prefectures, Japan.

Model [1] (SARIMA/ SARIMAX)	(p,d,q)(P,D,Q)S [2]	Coefficient [3] (95%CI)	AIC	Ljung-Box test [4]	RMSE (model fit)	RMSE (forecast)
Osaka						
SARIMA	(0,1,3)(2,0,0)12	NA	−72.97	0.47	0.132	0.113
Temperature	(0,1,1)(0,0,2)12	−0.022 (−0.026, −0.018)	−91.79	0.127	0.128	0.113
Humidity	(0,1,2)(2,0,0)12	−0.01 (−0.019, −0.001)	−75.96	0.446	0.131	0.137
Wind speed	(1,1,1)(2,0,0)12	−0.306 (−0.413, −0.199)	−96.11	0.79	0.117	0.104
Solar radiation	(3,1,2)(1,0,0)12	0.002 (−0.025, 0.029)	−63.73	0.141	0.144	0.163
Sunshine duration	(0,1,1)(1,0,0)12	0.061 (0.031, 0.091)	−70.45	0.143	0.136	0.178
NO	(0,0,1)(2,0,0)12	0.033 (0.027, 0.039)	−140.39	0.385	0.09	0.13
O_3	(0,1,3)(2,0,0)12	−0.002 (−0.009, 0.005)	−71.22	0.386	0.132	0.11
Yamanashi						
SARIMA	(4,0,0)(1,0,2)12	NA	−98.33	0.802	0.11	0.174
Temperature	(1,0,0)(0,1,1)12	−0.012 (−0.034, 0.010)	−113.25	0.591	0.091	0.102
Humidity	(1,0,1)(0,1,1)12	0.01 (0.006, 0.013)	−132.26	0.101	0.077	0.078
Wind speed	(0,0,3)(1,0,2)12	−0.198 (−0.251, −0.144)	−126.62	0.173	0.088	0.129
Solar radiation	(0,0,3)(0,1,1)12	−0.016 (−0.029, −0.004)	−117.52	0.552	0.086	0.097
Sunshine duration	(0,0,3)(0,1,1)12	−0.025 (−0.043, −0.006)	−117.6	0.432	0.085	0.1
NO	(0,0,3)(0,1,1)12	0.034 (0.021, 0.047)	−132.63	0.17	0.08	0.109
O_3	(0,0,3)(1,0,2)12	−0.007 (−0.012, −0.002)	−103.27	0.322	0.106	0.17
Tokyo						
SARIMA	(0,1,3)(2,0,1)12	NA	−125.11	0.753	0.094	0.116
Temperature	(0,1,2)(2,0,0)12	−0.022 (−0.029, −0.015)	−142.69	0.812	0.091	0.127
Humidity	(1,1,1)(1,0,0)12	0.004 (−0.001, 0.008)	−124.51	0.347	0.098	0.115
Wind speed	(1,0,1)(0,1,2)12	−0.181 (−0.242, −0.120)	−144.79	0.465	0.072	0.134
Solar radiation	(4,0,0)(0,1,1)12	−0.021 (−0.033, −0.009)	−128.95	0.974	0.081	0.119
Sunshine duration	(1,0,0)(1,0,0)12	−0.018 (−0.037, 0.001)	−125.99	0.288	0.1	0.108
NO	(0,1,2)(2,0,0)12	0.024 (0.016, 0.031)	−157.37	0.301	0.081	0.091
O_3	(1,1,1)(2,0,1)12	−0.003	−126.42	0.557	0.094	0.107

		(−0.009, 0.002)				
Aichi						
SARIMA	(1,0,1)(2,0,0)12	NA	−99.27	0.597	0.116	0.128
Temperature	(0,1,1)(2,0,0)12	−0.015 (−0.020, −0.009)	−107.73	0.663	0.114	0.111
Humidity	(1,0,1)(2,0,0)12	−0.003 (−0.010, 0.004)	−97.95	0.493	0.116	0.132
Wind speed	(0,0,2)(1,0,0)12	−0.27 (−0.343, −0.196)	−116.01	0.093	0.106	0.138
Solar radiation	(0,1,1)(2,0,0)12	−0.019 (−0.031, −0.007)	−105.87	0.554	0.111	0.107
Sunshine duration	(1,0,1)(2,0,0)12	−0.013 (−0.034, 0.008)	−98.66	0.599	0.114	0.128
NO	(1,0,0)(2,0,0)12	0.02 (0.013, 0.028)	−119.63	0.849	0.106	0.111
O_3	(1,0,1)(2,0,0)12	−0.001 (−0.008, 0.005)	−97.45	0.475	0.116	0.125

Notes: The first 7 years [April 2009–March 2016, 84-point (12 months times 7 years) time series data] were used as training data and the last 1 year [April 2016–March 2017, 12-point (12 months times 1 year) time series data] were as testing data for each model. SARIMA, seasonal autoregressive integrated moving average; SARIMAX, seasonal autoregressive integrated moving average with exogeneous variables; CI, confidence interval; AIC, Akaike information criteria; RMSE, root mean square error; NO_2, nitrogen dioxide; NO, nitric oxide; O_3, ozone. [1] This column shows the types of models. The exogeneous variables are shown for SARIMAX models. An exogeneous variable was included in each SARIMAX model. [2] (p,d,q)(P,D,Q)S, the number of autoregressive terms (p), differences (d), and moving average (q) for non-seasonal parameters, and the number of autoregressive terms (P), differences (D), and moving average for seasonal parameters in SARIMA a model. [3] Coefficients in log scale for exogeneous variable. [4] *p*-value for Ljung-Box test.

Table S3. Climate and air pollution factors associated with NO_2 concentration in the atmosphere from SARIMAX models at RAPMS in Tokyo, Aichi, Osaka, and Yamanashi Prefectures, Japan.

Model [1] (SARIMA/ SARIMAX)	(p,d,q)(P,D,Q)S[2]	Coefficient [3] (95%CI)	AIC	Ljung-Box test [4]	RMSE (model fit)	RMSE (forecast)
Tokyo						
SARIMA	(0,1,1)(0,0,2)12	NA	−149.8	0.561	0.087	0.095
Temperature	(0,1,1)(0,0,2)12	−0.001 (−0.005, 0.003)	−148.22	0.503	0.087	0.093
Humidity	(0,1,1)(0,0,2)12	0.002 (−0.001, 0.004)	−148.99	0.338	0.087	0.093
Wind speed	(0,1,1)(0,1,1)12	−0.297 (−0.344, −0.251)	−198.82	0.335	0.05	0.074
Solar radiation	(0,1,1)(0,0,2)12	−0.002 (−0.01, 0.005)	−148.14	0.435	0.087	0.092
Sunshine duration	(0,1,1)(0,0,2)12	−0.021 (−0.039, −0.003)	−152.94	0.22	0.086	0.095
NO	(1,0,0)(2,0,0)12	0.006 (0.005, 0.008)	−178.07	0.534	0.075	0.098
AO	(0,1,1)(2,0,0)12	−0.003 (−0.012, 0.001)	−152.3	0.387	0.085	0.112
Aichi						
SARIMA	(0,0,3)(2,0,2)12	NA	−115.21	0.096	0.096	0.142
Temperature	(0,1,1)(2,0,0)12	−0.016 (−0.022, −0.009)	−132.72	0.301	0.093	0.111
Humidity	(1,0,0)(2,0,1)12	−0.005 (−0.010, 0.000)	−122.89	0.09	0.095	0.135
Wind speed	(0,0,0)(1,1,0)12	−0.169 (−0.238, −0.100)	−138.02	0.124	0.076	0.109
Solar radiation	(0,0,0)(1,1,0)12	−0.002 (−0.014, 0.011)	−118.44	0.27	0.088	0.105
Sunshine duration	(0,0,0)(1,1,0)12	−0.002 (−0.020, 0.016)	−118.41	0.291	0.088	0.105
NO	(0,0,0)(2,0,0)12	0.009 (0.007, 0.012)	−162.66	0.515	0.08	0.088
AO	(0,1,1)(1,1,0)12	0.001 (−0.005, 0.007)	−112.4	0.246	0.089	0.12
Osaka						
SARIMA	(0,1,3)(2,0,0)12	NA	−112.47	0.543	0.109	0.071
Temperature	(2,1,3)(0,0,0)12	−0.011 (−0.013, −0.008)	−123.83	0.49	0.103	0.084
Humidity	(0,1,3)(2,0,0)12	−0.006 (−0.012, −0.001)	−115.12	0.683	0.106	0.079
Wind speed	(0,1,3)(2,0,0)12	−0.257 (−0.317, −0.198)	−146.11	0.268	0.084	0.067
Solar radiation	(0,1,3)(2,0,0)12	0.01 (−0.006, 0.026)	−111.86	0.56	0.106	0.078
Sunshine duration	(0,1,3)(2,0,0)12	0.025 (0.003, 0.047)	−114.88	0.566	0.104	0.077
NO	(0,0,4)(2,0,0)12	0.015 (0.012, 0.018)	−166.76	0.295	0.074	0.05
AO	(3,1,1)(2,0,0)12	−0.006	−113.43	0.38	0.103	0.084

		(−0.011, −0.001)				
Yamanashi						
SARIMA	(0,0,2)(2,0,0)12	NA	−71.46	0.502	0.14	0.234
Temperature	(4,1,2)(1,0,0)12	−0.031<br>(−0.038, −0.024)	−83.52	0.659	0.129	0.21
Humidity	(2,0,0)(2,0,0)12	0.005<br>(0.001, 0.010)	−73.63	0.216	0.139	0.24
Wind speed	(2,0,0)(2,0,0)12	−0.12<br>(−0.208, −0.032)	−75.66	0.114	0.138	0.24
Solar radiation	(1,1,4)(2,0,0)12	−0.005<br>(−0.022, 0.012)	−68.75	0.371	0.136	0.186
Sunshine duration	(0,0,2)(2,0,0)12	0.012<br>(−0.008, 0.032)	−70.86	0.381	0.139	0.233
NO	(1,0,1)(2,0,0)12	0.038<br>(0.025, 0.050)	−98.5	0.832	0.12	0.2
AO	(2,0,0)(2,0,0)12	−0.009<br>(−0.015, −0.005)	−82.59	0.673	0.131	0.229

Notes: The first 7 years [April 2009–March 2016, 84-point (12 months times 7 years) time series data] were used as training data and the last 1 year [April 2016–March 2017, 12-point (12 months times 1 year) time series data] were as testing data for each model. SARIMA, seasonal autoregressive integrated moving average; SARIMAX, seasonal autoregressive integrated moving average with exogeneous variables; CI, confidence interval; AIC, Akaike information criteria; RMSE, root mean square error; NO_2, nitrogen dioxide; NO, nitric oxide; AO, active ozone. [1] This column shows the types of models. The exogeneous variables are shown for SARIMAX models. An exogeneous variable was included in each SARIMAX model. [2] (p,d,q)(P,D,Q)S, the number of autoregressive terms (p), differences (d), and moving average (q) for non-seasonal parameters, and the number of autoregressive terms (P), differences (D), and moving average for seasonal parameters in SARIMA a model. [3] Coefficients in log scale for exogeneous variable. [4] *p*-value for Ljung-Box test.
